# Items

**Published:** 1883-03

**Authors:** 


### Items.

METEOROLOGICAL DATA.—For the month of Jan., 1883. H. C. Smyth, Observer, U. S. Signal Station, Atlanta, Ga.

| | | |
|---|---|---|
| BAROMETER | —Mean | 30.217 |
| | Highest | 30.667 on 23rd. |
| | Lowest | 29.745 on 9th. |
| | Range | .922 |
| THERMOMETER | —Mean | 49.6 |
| | Highest | 63.8 on 28th. |
| | Lowest | 14.2 on 12th. |
| | Range | 49.6 |
| | Greatest Daily Range | 37.7 on 21st. |
| | Least Daily Range | 5.0 on 16th. |
| Humidity | —Mean | 77.8 |
| Dew-point | —Mean | 35.1 |
| Rainfall | —Total | 15.82 inches. |
| | Greatest Daily | 4.03 on 6th. |
| | Total Number of Days | 20 |
| Wind | —Prevailing Direction | N. W. |
| | Maximum Velocity | W., 34 miles per hour. |

THE latest governor which Massachusetts has produced is opposed to some of the charitable institutions of that

State. In his inaugural, if correctly reported, he declares that "the school for idiotic and feeble-minded youth ought to be swept out of existence, since a well-fed, well-cared for idiot is a happy creature, while an idiot awakened to his condition is a miserable one."

This, no doubt, represents that man's idea of reform, and is a just measure of his capacity to estimate the value of such institutions as he decries. It is well understood that the reason he assigns for his recommendation is untenable, but a person capable of brutality toward defenseless women could scarcely be expected to entertain humane and tender feelings for helpless children.

---

THE BIRMINGHAM, (England), *Medical Review*, warmly indorses Dr. George W. Noble's devise for the treatment of mammitis which was published in the REGISTER a few months ago. After a trial upon two cases, the editor unreservedly recommends it to the gynecologists and surgeons of Great Britain, as worthy of confidence and as a valuable surgical appliance in appropriate cases.

---

THE Committee of the American Medical Association, having under consideration the subject of establishing a weekly journal similar to the *British Medical Journal*, met in Chicago, January 17th, and decided that the publication of a journal by the Association is warranted. It will be located in Chicago and Dr. N. S. Davis will be editor-in-chief.

---

DR. JOHN THAD. JOHNSON is still confined to his home in consequence of the extreme illness noted in our last issue. While no radical change in his condition can be chronicled at this date, his general symptoms, for several days past, lead his friends to hope for a favorable progress in his case henceforth.

---

DEATH has not spared the profession of late. Among the prominent victims of fatal diseases are the following well known physicians: J. Forsyth Meigs, of Philadelphia; Sir Thomas Watson, England; W. M. Charters, Savannah, and George M. Beard, New York.

